# Impact of irradiation on the reproductive traits of field and laboratory *An. arabiensis* mosquitoes

**DOI:** 10.1186/s13071-018-3228-3

**Published:** 2018-12-17

**Authors:** Serge B. Poda, Edwige Guissou, Hamidou Maïga, Sévérin N. Bimbile-Somda, Jérémie Gilles, Jean-Baptiste Rayaisse, Thierry Lefèvre, Olivier Roux, Roch K. Dabiré

**Affiliations:** 1 Institut de Recherche en Sciences de la Santé (IRSS), Bobo-Dioulasso 01, 01 BP 545 Burkina Faso; 2 MIVEGEC, IRD, CNRS, University of Montpellier, Montpellier, France; 3 Joint FAO/IAEA Division of Nuclear Techniques in Food and Agriculture, Insect Pest Control Laboratory, Vienna, Austria; 4 Centre International de Recherche-Développement sur l’Elevage en zone Subhumide (CIRDES), Bobo-Dioulasso 01, 01 BP 454 Burkina Faso

**Keywords:** Egg retention, Fecundity, Fertility, Insemination, Longevity, Malaria vector, Oviposition, Sterile insect technique

## Abstract

**Background:**

The sterile insect technique (SIT) aims at suppressing or decreasing insect pest populations by introducing sterile insects into wild populations. SIT requires the mass-production of insects and their sterilization through, for example, radiation. However, both mass-rearing and radiation can affect the life history traits of insects making them less competitive than their wild counterparts. In the malaria mosquito *Anopheles arabiensis*, some progress has been made to improve the mating competitiveness of mass-reared irradiated males. However, to date, no study has explored the relative effects of colonization and irradiation on important reproductive traits in this species. Such data may help to focus research efforts more precisely to improve current techniques.

**Methods:**

Two strains of *An. arabiensis* originating from the same locality were used: one reared in the laboratory for five generations and the second collected as late larval instars in the field prior to experimentation. Pupae were irradiated with 95 Gy and some adult reproductive traits, including insemination rate, fecundity, oviposition behavior, fertility and male survivorship, were assessed in different mating combinations.

**Results:**

Our study revealed the different effects of mosquito strain and irradiation on reproductive processes. The insemination rate was higher in field (67.3%) than in laboratory (54.9%) females and was negatively affected by both female and male irradiation (un-irradiated *vs* irradiated: 70.2 *vs* 51.3% in females; 67.7 *vs* 53.7% in males). Irradiated females did not produce eggs and egg prevalence was lower in the field strain (75.4%) than in the laboratory strain (83.9%). The hatching rate was higher in the field strain (88.7%) than in the laboratory strain (70.6%) as well as in un-irradiated mosquitoes (96.5%) than in irradiated ones (49%). Larval viability was higher in the field strain (96.2%) than in the laboratory strain (78.5%) and in un-irradiated mosquitoes (97.6%) than irradiated ones (52%). Finally, field males lived longer than laboratory males (25.1 *vs* 20.5 days, respectively).

**Conclusions:**

Our results revealed that both irradiation and colonization alter reproductive traits. However, different developmental stages are not equally affected. It is necessary to consider as many fitness traits as possible to evaluate the efficacy of the sterile insect technique.

**Electronic supplementary material:**

The online version of this article (10.1186/s13071-018-3228-3) contains supplementary material, which is available to authorized users.

## Background

Faced with the increase in insecticide resistance, it is necessary to design new tools or improve existing methods to better control vector-borne diseases. During the past decade, there has been renewed interest in the use of the sterile insect technique (SIT) which aims to suppress or decrease insect pest populations by introducing sterile insects into wild populations. The principle is based on the insemination of wild females by sterile, laboratory-reared males resulting in non-viable progeny and hence in a reduction in pest populations [1]. To reach this goal, the SIT requires the mass production of insects, sterilization, gender separation, and the transportation and release of males only into the target areas [2, 3]. Among the techniques available or under investigation to induce sterility (see [4] for an overview), the use of radiation for SIT is recognized as the safest for human health, is environmentally friendly, species-specific, and does not require regulation for use in the field [2, 5, 6]. Moreover, it has proved its efficacy in the field on various insect pest species [1].

To be efficient, the SIT requires releasing a large number of laboratory-reared males which have to be as competitive as wild males to mate with wild females. However, irradiation and insect mass production can reduce the competitiveness of sterile males in several ways [7, 8]. First, radiation induces an oxidative stress which can affect germ cells through lethal DNA mutations. The higher the radiation dose, the higher the rate of mutation. These mutations do not lead to cell death and do not affect gamete or zygote formation but can cause the failure of embryo development [9]. Secondly, oxidative stress can directly affect somatic cells. DNA, protein and lipid oxidation alter cells, tissues and organism functioning which can lead to changes in life history traits [10]. Reduced longevity is the most evident effect of a high irradiation level but sperm production, emergence success, courtship behavior, pheromone production, and the structure of flight muscles can also be affected [7, 8]. Finally, insect colonization and mass rearing over many generations can cause the loss of natural genetic traits. Indeed, as only a fraction of field individuals survive during colonization, laboratory populations undergo genetic bottlenecks resulting in the selection of traits more adapted to insectary conditions [8, 11]. Many reproductive traits such as fecundity, courtship, oviposition, the production of and response to pheromones [11–14], but also other traits involved in mating or oviposition behavior such as flight ability, eye morphology, visual sensitivity and resistance to stress [15–17] have been described as being affected and possibly reducing overall insect vigor in the wild.

Despite recent improvements in both irradiation and laboratory mass-rearing techniques, SIT for *Anopheles* mosquito species has not reached an operational level as it has for some other pest insects [18]. *Anopheles arabiensis* is one of the main malaria vectors in Africa. Recent studies have provided valuable information on its radiation biology including the effects of variable radiation doses [19, 20] and mosquito developmental stages that should be targeted for irradiation [20–22]. While the impact of radiation on sperm production [23, 24], male competitiveness [19, 21, 25], longevity [22], fertility and fecundity [26] was previously assessed in laboratory conditions, some field and semi-field trials investigated the impact of transportation on mortality [27], the participation of sterile males in swarms and their dispersion capacities [28, 29]. To date, however, there is little information on the relative effects of radiation and laboratory rearing on the life history traits of *An. arabiensis*. Here we compare the effect of irradiation on the reproductive traits of laboratory *vs* wild *An. arabiensis* from the same locality.

## Methods

### Mosquitoes

Both field and laboratory *An. arabiensis* mosquitoes were used and originated from Dioulassoba, a central urban area of Bobo-Dioulasso, Burkina Faso. The strain considered as “laboratory” mosquitoes was established from wild gravid *An. arabiensis* females collected indoors in 2015. Females were placed individually in oviposition cups containing tap water. After oviposition, the females were all identified as *An. arabiensis* using PCR as described by Fanello et al. [30]. The larvae were reared in tap water exposed to controlled conditions in the insectaries (27 ± 2 °C, 70 ± 10% RH, 12L:12D) and fed with Tetramin® Baby Fish Food (Tetrawerke, Melle, Germany) *ad libitum*. Females were blood-fed on rabbit for egg production and reared in insectary conditions. Experiments were conducted in 2016 with the fifth generation of the laboratory strain and with F0 individuals that emerged from wild larvae collected in 2016 (“field” strain).

### Mosquito irradiation

Prior to irradiation, *ca.* 20-h-old pupae were randomly assigned to two different plastic cups (Ø = 45 mm, h = 85 mm) at similar densities: an irradiated group and a control group. To ensure uniform radiation dose distribution, pupal densities did not exceed 200 pupae per cup. Only 1cm of water was left to limit radiation absorbance by the water. Pupae were irradiated at a theoretical dose of 92 Gy in a Gamma Cell ^137^Cs self-contained gamma source at a rate of about 4 Gy/min for 23 min. This dose has been shown to allow a good sterility-competitiveness trade-off in *An. arabiensis* males [22]. The cups were placed at the centre of the chamber to maximize dose uniformity within the batch. A dosimetry system was used to measure the accurate dose received by each batch using Gafchromic® HD-V2 film (Ashland, Bridgewater, NJ, USA) placed on the walls of the cups. After irradiation, the optical density of the irradiated films was read at both 458 and 590 nm with a dose reader (Dosereader4, Radgen, Budapest, Hungary) and compared to a control. The dose effectively measured was 95.4 ± 0.9 Gy (mean ± standard error, SE). The control group was handled similarly but was not irradiated. Following irradiation, irradiated and control pupae were placed in 30 × 30 × 30 cm mesh-covered cages separately and kept under standard insectary conditions (27 ± 2 °C; 70 ± 10% RH; 12L:12D photoperiod). The next day, emerged mosquitoes were separated by gender early in the morning and males and females were kept in separate 30 × 30 × 30 cm cages to prevent mating. They were maintained under insectary conditions and provided with a 5% glucose solution. The number of dead pupae was recorded. Three to four batches of mosquitoes were irradiated on consecutive days for each replicate in order to obtain enough mosquitoes for experiments.

### Mosquito traits

A series of mosquito traits playing key roles in determining sexual competitiveness was measured, namely insemination rate, fecundity, fertility and longevity. The insemination rate was defined as the proportion of females with spermatozoa-positive spermathecae. Fecundity was defined as the capacity of females to produce eggs and fertility as their capacity to produce viable offspring. Longevity was considered as the lifespan from the first day in mating cages to death. To gauge the effect of irradiation on these traits, 2-day-old males and females (un-irradiated and irradiated) were assorted to obtain four mating combinations for each mosquito strain (laboratory *vs* field) as follows: both un-irradiated males and females (un-irradiated pairs, hereafter), irradiated males and un-irradiated females, un-irradiated males and irradiated females and both irradiated males and females (irradiated pairs, hereafter) (Additional file 1: Table S1). Males and females were kept together in 20 × 20 × 20 cm cages and provided with a 5% glucose solution for two nights. For each mating combination, two to three cages were used. A mating ratio of two males for one female was used with 40 to 80 males per cage.

On the second night, all of the females were removed from the cages and put into cardboard cups (Ø = 75 mm, h = 100 mm) for fecundity and fertility assays. The males were kept in their cages and provided with a 2.5% glucose solution every other day and water *ad libitum* every day. Male longevity was assessed by counting and then removing dead individuals every 24 h. To gauge the effect of irradiation on fecundity and fertility, females were provided with two blood meals on a rabbit at two-day intervals. Females that had not fed were discarded after each blood meal. Blood-fed females were kept in the cardboard cups and provided with a 5% glucose solution. Three days after the second blood meal, the females were placed individually in plastic oviposition cups (Ø = 45 mm, h = 85mm) containing tap water. Following egg laying, the spermatheca was dissected and the insemination status assessed under a microscope at 400× magnification to observe the presence/absence of spermatozoa. Females that did not lay eggs after 10 days were killed and their spermatheca dissected. The ovaries of all of the females were also dissected and the presence/absence of eggs as well as the number of unlaid eggs, if any, were recorded. Laid eggs were kept in plastic cups containing water for one week to allow them to hatch and the presence/absence of viable larvae was recorded. Then, hatched and unhatched eggs were counted to assess fertility. For both field and laboratory mosquitoes, two replicates were performed.

### Statistical analysis

All analyses were conducted in R v.2.12.1. The effect of irradiation and mosquito strain on a series of mosquito traits was analyzed using different statistical models summarized in Additional file 1: Table S2. These traits were the emergence rate of adult mosquitoes, female insemination rate (the contents of the spermatheca, i.e. presence/absence of spermatozoa), egg prevalence (the proportion of females that developed eggs in their ovaries), egg load (the number of eggs in gravid females), oviposition rate (the proportion of females that laid at least one egg), laid egg proportion (the number of eggs laid out of the total number of eggs produced), hatching rate (the proportion of egg batches in which at least one egg hatched), hatching proportion (the proportion of eggs that hatched within each egg batch), viable larvae prevalence (the proportion of egg batches in which at least one larva survived more than 3 days) and male survivorship (the length of time between the first day in mating cages and death). The general building of the statistical models was as follows. Mosquito strain (laboratory and field), treatment (irradiated and un-irradiated), insemination status (inseminated and non-inseminated) and their interactions were considered fixed effects. Plastic cups and mosquito cages were considered random effects.

For model selection, we used the stepwise removal of terms, followed by likelihood ratio tests. Term removals that significantly reduced explanatory power (*P* < 0.05) were retained in the minimal adequate model [31].

## Results

### Emergence rate

A total of 2420 and 3000 pupae were obtained from the field and the laboratory populations, respectively. Half of each group was irradiated (i.e. 1210 and 1500 pupae, respectively); the other halves were used as un-irradiated controls. The mean emergence rate (± 95% CI) was 89.5 ± 1.2% and was not affected by irradiation (un-irradiated: 89.9 ± 1.7% *vs* irradiated: 89 ± 1.8%), mosquito strain (field: 90.8 ± 1.3% *vs* laboratory: 87.6 ± 2.2%) or their interaction (Table 1).

**Table 1:** Effects of mosquito strain, irradiation and insemination status on reproductive traits and survivorship

Factors	*χ* ^2^	*df*	*P*-value
Emergence rate			
Strain	1.90	1	0.167
Treatment	0.32	1	0.568
Strain: Treatment	0.07	1	0.784
Insemination rate			
Strain	**7.83**	**1**	**0.005**
Male treatment	**9.69**	**1**	**0.001**
Female treatment	**18.16**	**1**	**<0.001**
Strain: Male treatment	1.45	1	1.227
Strain: Female treatment	0.93	1	0.334
Egg prevalence			
Strain	**5.45**	**1**	**0.019**
Treatment^a^	0.25	1	0.611
Insemination status	**24.54**	**1**	**<0.001**
Strain: Treatment	0.00	1	0.994
Strain: Insemination status	1.88	1	0.170
Egg load			
Strain	0.48	1	0.480
Treatment	0.96	1	0.327
Insemination status	**5.42**	**1**	**0.019**
Strain: Treatment	1.36	1	0.243
Strain: Insemination status	0.04	1	0.841
Treatment: Insemination status	2.58	1	0.108
Oviposition rate			
Strain	**9.70**	**1**	**0.001**
Treatment	0.56	1	0.452
Strain: Treatment	0.02	1	0.879
Laid egg proportion			
Strain	**4.68**	**1**	**0.030**
Treatment	1.41	1	0.233
Strain: Treatment	0.16	1	0.688
Hatching rate			
Strain	**5.10**	**1**	**0.023**
Treatment	**17.11**	**1**	**<0.001**
Strain: Treatment	0.98	1	0.321
Hatching proportion			
Strain	**5.44**	**1**	**0.019**
Treatment	**64.36**	**1**	**<0.001**
Strain: Treatment	1.79	1	0.180
Larval prevalence			
Strain	**4.83**	**1**	**0.027**
Treatment	**12.35**	**1**	**<0.001**
Strain: Treatment	1.66	1	0.196
Survivorship			
Strain	**8.21**	**1**	**0.004**
Treatment	0.54	1	0.458
Strain: Treatment	0.08	1	0.772

Strain (field or laboratory), treatment (un-irradiated or irradiated) and insemination status (inseminated or not)

^a^As irradiated females did not produce eggs, only the treatment of males was considered (starting with egg prevalence and below), bold values are significant

### Insemination rate

**Fig. 1:**
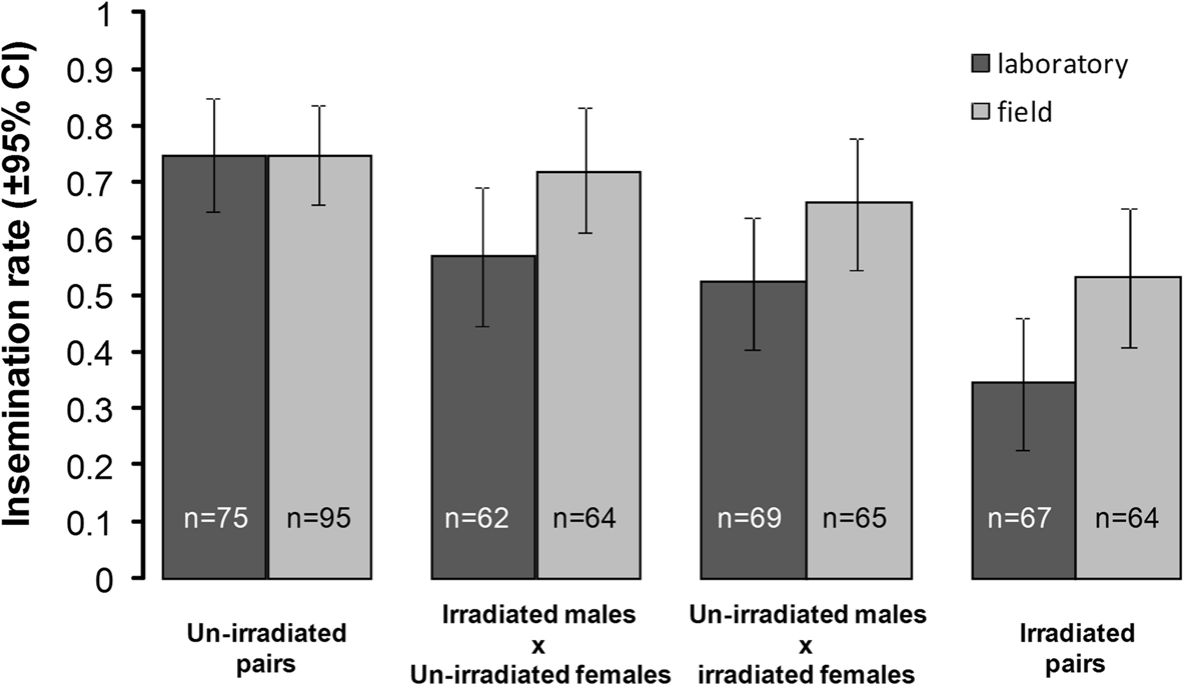
Effects of mosquito strain and irradiation on female insemination rate. The female insemination rate was assessed after two mating nights for the four mating pair combinations. The numbers in the bars indicate sample size

A significant negative effect of male and female irradiation on insemination was found (un-irradiated and irradiated: 67.7 ± 5.2% *vs* 53.7 ± 6.1% in males and 70.2 ± 6% *vs* 51.3 ± 5.2% in females). The insemination rate was significantly higher in field females than in laboratory females (67.3 ± 5.4% *vs* 54.9 ± 5.9%). There was no interaction between irradiation and mosquito strain (Fig. 1 and Table 1).

### Egg prevalence

**Fig. 2:**
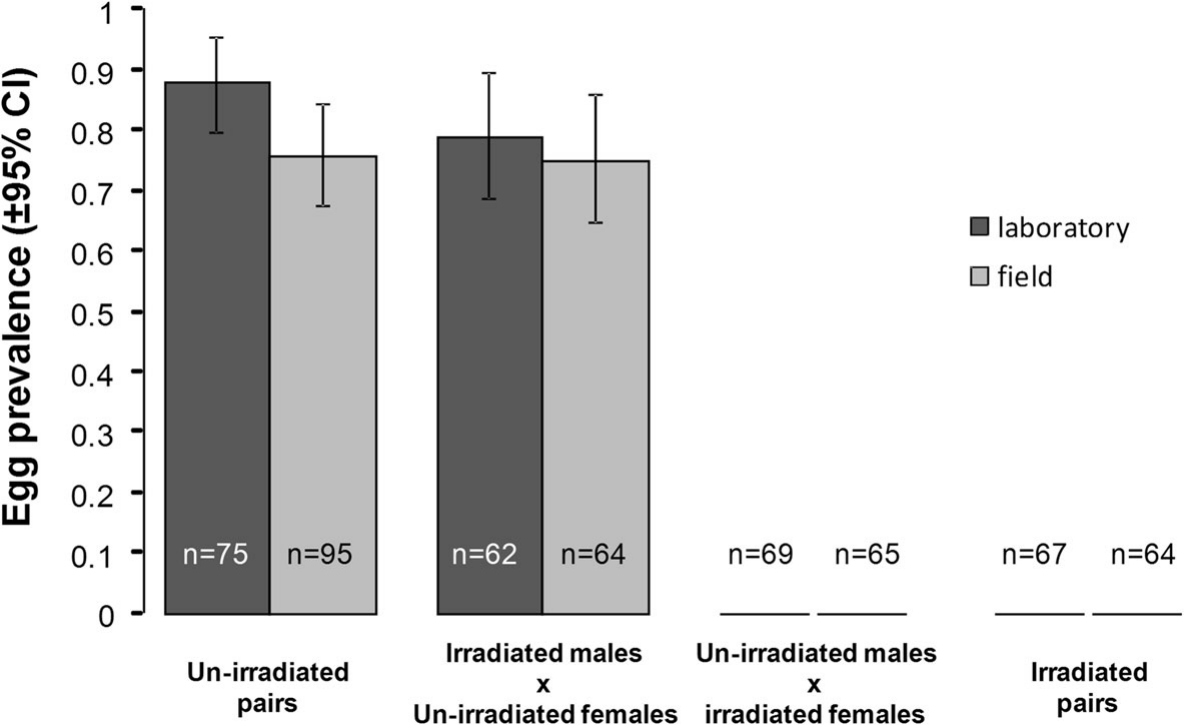
Effects of mosquito strain and irradiation on egg prevalence. Egg prevalence is assessed through females that had at least one egg in their ovaries or laid at least one egg. The numbers in the bars indicate sample size

Egg prevalence was defined as females that had at least one egg in their ovaries or that had laid at least one egg after two blood meals. We found a significant negative effect of irradiation on females with irradiated females never producing eggs (Fig. 2). The analysis conducted on un-irradiated females only showed that there was a significant effect of female insemination status on egg prevalence with non-inseminated females being less likely to produce eggs than inseminated females (61.3 ± 10.1% *vs* 87 ± 4.5%, respectively; Fig. 2, Table 1). There was a significant effect of mosquito strain on egg prevalence with un-irradiated field females being less likely to produce eggs than un-irradiated laboratory females (75.4 ± 6.6% *vs* 83.9 ± 6.1%, respectively; Fig. 2, Table 1). There was no interaction between irradiation and mosquito strain (Fig. 2, Table 1) or between strain and insemination status (Table 1).

### Egg load

**Fig. 3:**
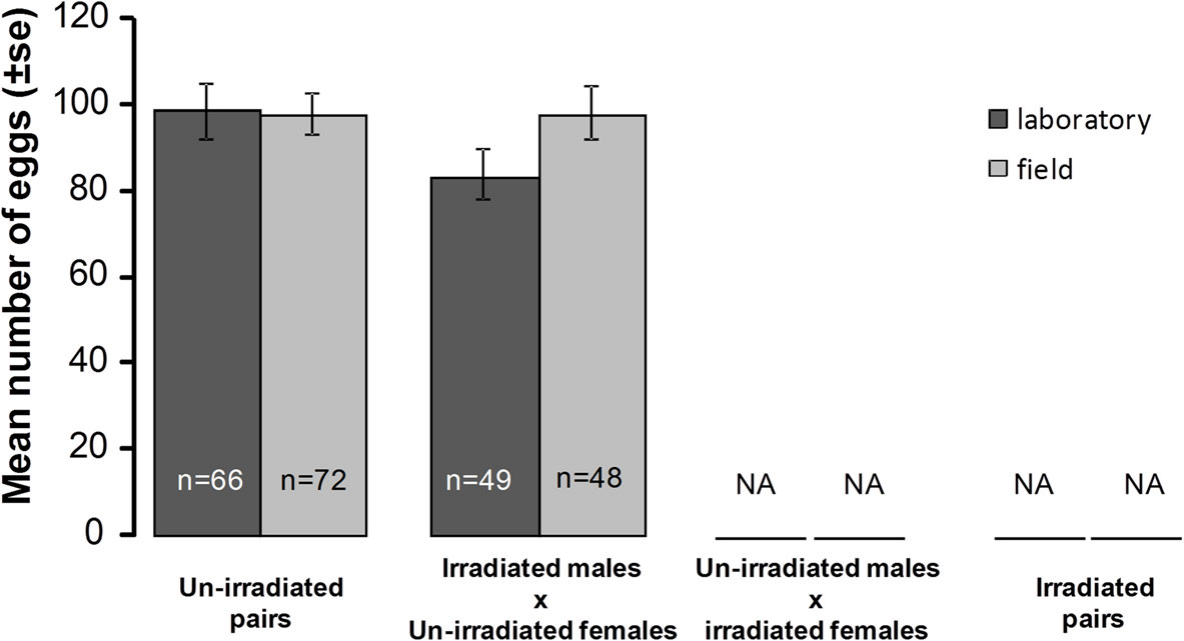
Effects of mosquito strain and irradiation on mean number of eggs in un-irradiated, gravid females. The number of eggs is the sum of eggs laid or retained in the ovaries per female. The numbers in the bars indicate sample size. NA: no data as irradiated females did not produce eggs

In un-irradiated gravid females, the mean egg load (± SE) (i.e. the number of laid eggs and/or eggs retained in the ovaries) was not affected by male irradiation (un-irradiated: 98.7 ± 3.9 *vs* irradiated: 91.3 ± 4.3 eggs) or by mosquito strain (field: 98.5 ± 3.7 *vs* laboratory: 92.7 ± 4.5 eggs; Fig. 3, Table 1). Egg load was, however, affected by insemination status with inseminated females having significantly more eggs than non-inseminated ones (99.6 ± 3.2 *vs* 82.5 ± 6.5 eggs, respectively; Table 1, Additional file 1: Figure S1). There was no interaction between irradiation status and mosquito strain (Fig. 3, Table 1), irradiation status and insemination status or between mosquito strain and insemination status (Table 1).

### Oviposition rate

**Fig. 4:**
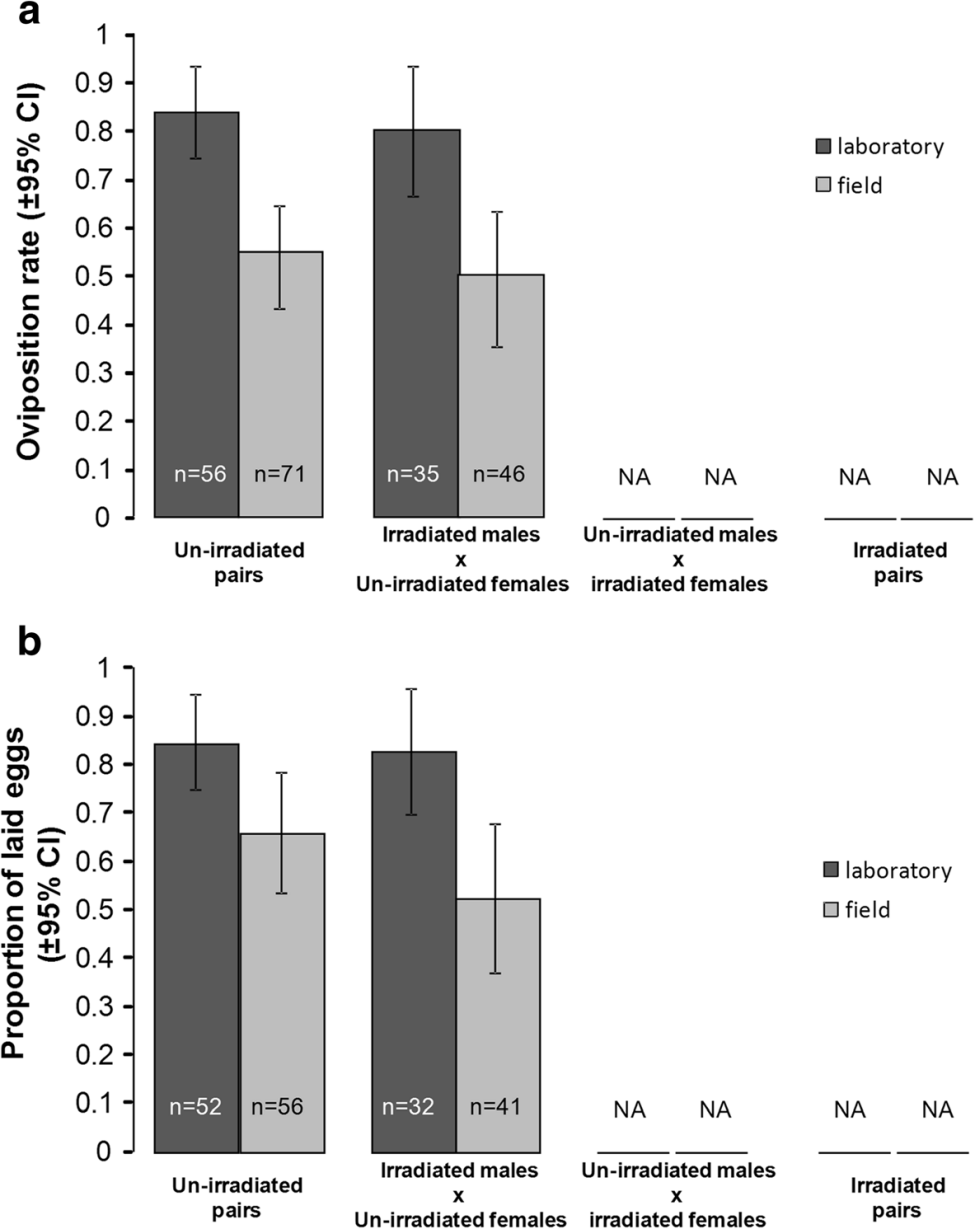
Effects of mosquito strain and male irradiation on un-irradiated female oviposition behavior: **a** oviposition rate (i.e. females that laid at least one egg) and **b** proportion of eggs laid per female [number of eggs laid out of the total number of eggs produced (laid eggs + retained eggs) per female]. The numbers in the bars indicate sample size. NA: no data as irradiated females did not produce eggs

The oviposition rate (i.e. females that had laid at least one egg) was affected by insemination status, with non-inseminated females never laying eggs (Additional file 1: Figure S2). As non-inseminated and irradiated females never laid eggs, we analyzed the oviposition rate on un-irradiated gravid and inseminated females only. Insemination by irradiated males had no effect on the oviposition rate (un-irradiated: 67.7 ± 8.1% *vs* irradiated: 62.9 ± 10.5%; Fig. 4a, Table 1). However, laboratory females were more likely to lay eggs than were their field counterparts (82.4 ± 7.8% *vs* 52.9 ± 9%, respectively; Fig. 4a, Table 1). There was no interaction between male irradiation status and mosquito strain (Fig. 4a, Table 1).

### Laid egg proportion

Gravid females did not lay their eggs all at once. The proportion of eggs laid per female was significantly affected by mosquito strain with field females retaining eggs more frequently than laboratory females (proportion of eggs laid: 60.1 ± 7.1% *vs* 83.7 ± 5.3%, respectively; Fig. 4b, Table 1). Insemination by irradiated males had no effect on egg retention (un-irradiated: 74.8 ± 6.3% *vs* irradiated: 65.6 ± 6.9%; Fig. 4b, Table 1). There was no interaction between male irradiation and mosquito strain (Fig 4b, Table 1).

### Hatching rate

**Fig. 5:**
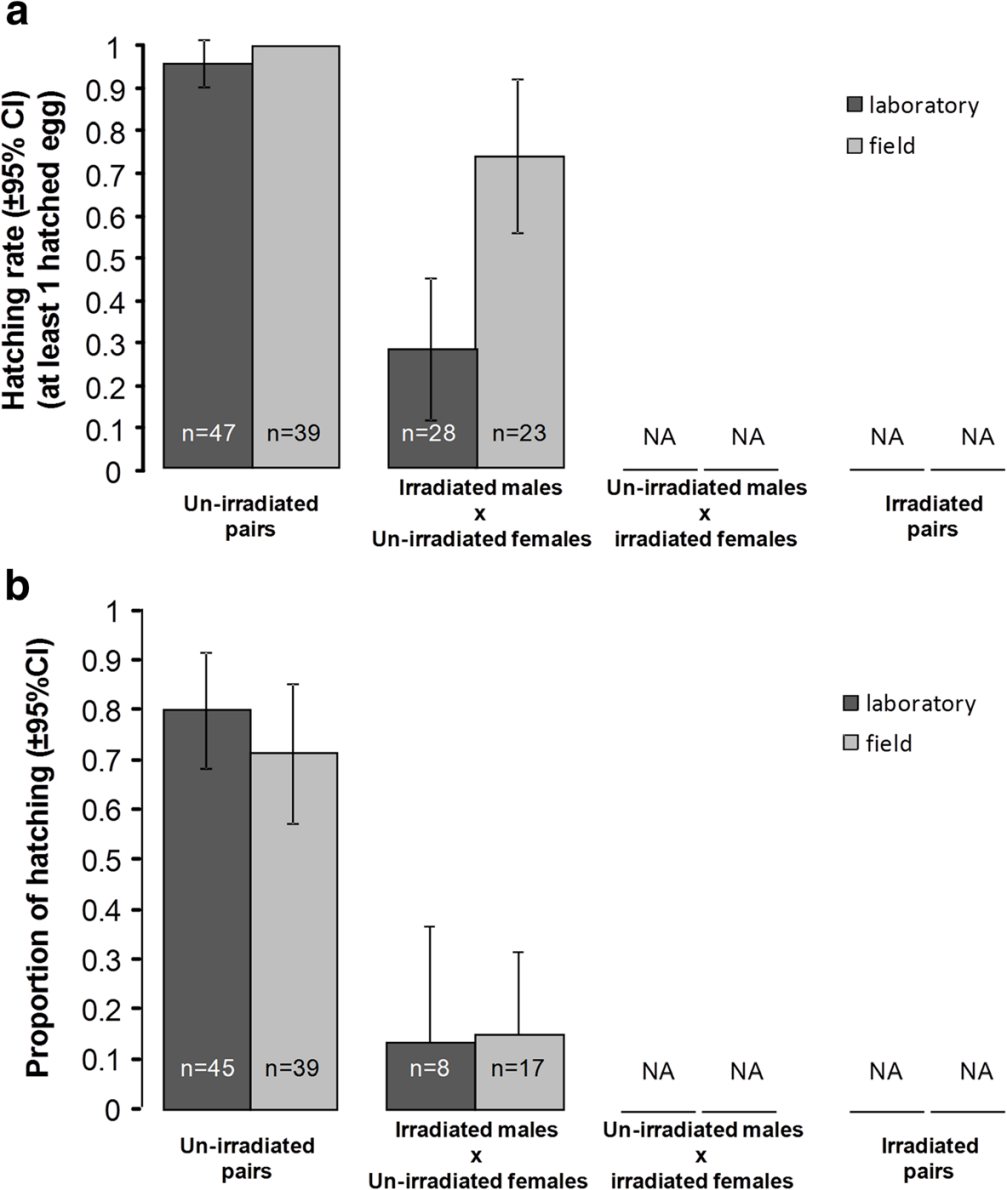
Effects of mosquito strain and male irradiation on egg hatching. **a** Hatching rate per egg batch (i.e. presence of at least one hatched egg in the batch). **b** Hatching proportion within egg batches (i.e. number of hatched eggs over the total number of eggs laid per batch). The numbers in the bars indicate sample size. NA: no data as irradiated females did not produce eggs

Hatching rate (i.e. presence of at least one hatched egg in the batches) was negatively affected by insemination by an irradiated male (un-irradiated: 96.5 ± 3.8% *vs* irradiated: 49 ± 13.7%; Fig. 5a, Table 1) and by mosquito strain with field mosquitoes having better hatching success than their laboratory counterparts (field: 88.7 ± 7.8% *vs* laboratory: 70.6 ± 10.3%; Fig. 5a, Table 1). There was no interaction between male irradiation and mosquito strain (Fig. 5a, Table 1).

### Hatching proportion

To gauge more precisely the reproduction potential of partially sterilized males, we also considered the proportion of hatching within egg batches (i.e. the number of hatched eggs out of the total number of eggs laid per batch). Hatching proportion was negatively affected by male irradiation (un-irradiated: 76.1 ± 8% *vs* irradiated: 14.3 ± 6.6%) and eggs from laboratory mosquitoes hatched more frequently than those from field mosquitoes (field: 54 ± 9.3% *vs* laboratory: 70 ± 8.6%; Fig. 5b, Table 1). There was no interaction between irradiation and mosquito strain (Fig. 5b, Table 1).

### Larval prevalence

**Fig. 6:**
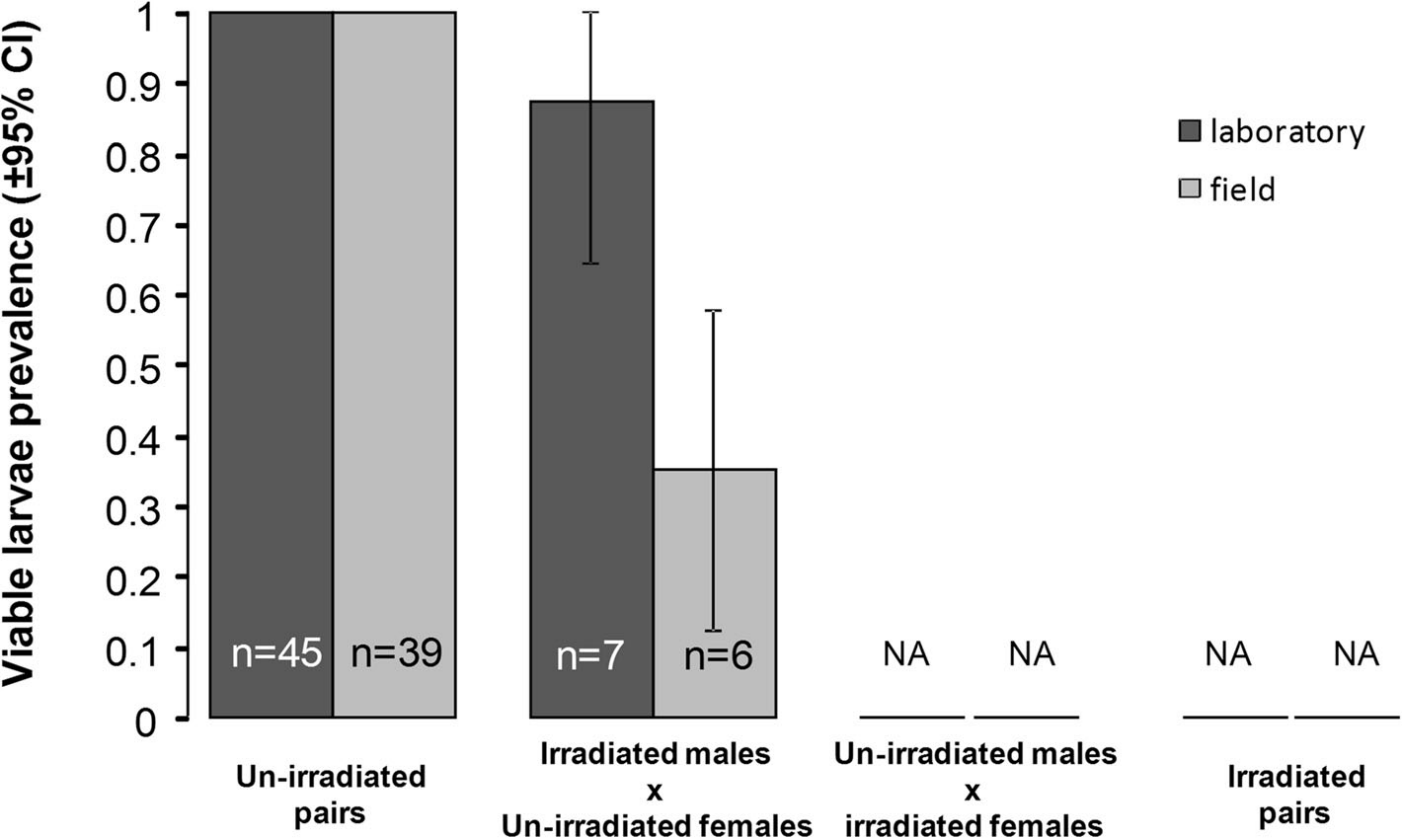
Effects of mosquito strain and male irradiation on first-instar larva viability. The numbers in the bars indicate sample size. NA: no data as irradiated females did not produce eggs

Male irradiation also reduced the prevalence of viable larvae (fertility) (un-irradiated: 97.6 ± 3.2% *vs* irradiated: 52 ± 19.5%). Larval viability was also higher in laboratory than in field mosquitoes (field: 78.5 ± 10.7% *vs* laboratory: 96.2 ± 5.1%; Fig. 6, Table 1). There was no interaction between male irradiation and mosquito strain (Fig. 6, Table 1).

### Longevity

**Fig. 7:**
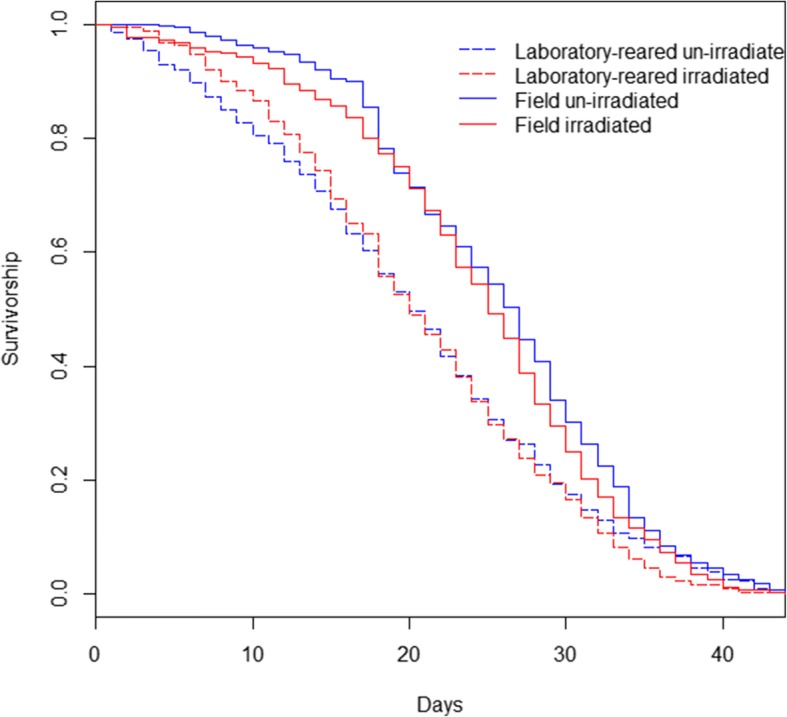
Effects of mosquito strain and irradiation on male survivorship

Male survivorship was not influenced by irradiation (un-irradiated: 22.8 ± 0.3 *vs* irradiated: 22.3 ± 0.3 days). However, field males lived longer than those reared in the laboratory (field: 25.1 ± 0.3 *vs* laboratory: 20.5 ± 0.3 days; Fig. 7, Table 1). There was no interaction between irradiation and mosquito strain (Fig. 7, Table 1).

**Fig. 8:**
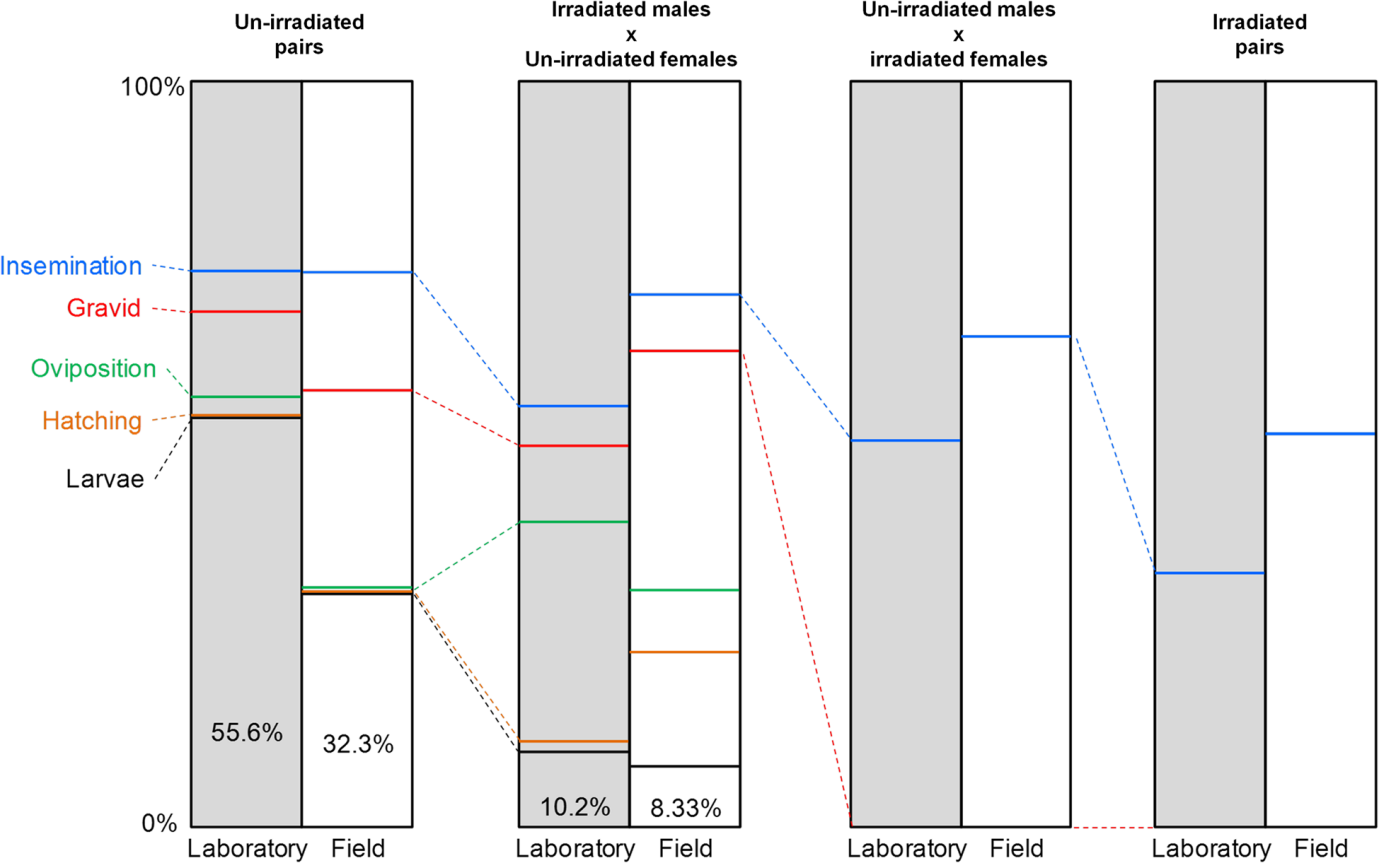
Fertility potential in mating pairs for laboratory and field mosquitoes. 100% represents the full fertility potential and corresponds to the situation in which all the females of a theoretical population give birth to at least one viable larva. “Insemination” is the rate of insemination in the population. “Gravid” is the proportion of females that are gravid among those that are inseminated. “Oviposition” is the proportion of females that succeed in laying eggs among the females that are both inseminated and gravid. “Hatching” is the proportion of egg batches in which at least one egg hatched. “Larvae” is the proportion of egg batches that result in at least one viable larva. The numbers in the columns are the proportions of females that have at least one offspring (i.e. realized fertility *vs* overall population sterility). Each interval between two traits represents the proportion of females that failed to reach the stage in the reproductive process being considered. All proportions were obtained from our experiments

Figure 8 shows an attempt to estimate the theoretical “realized fertility” rates in females based on our results. It shows that mating pairs including irradiated females had a null fertility rate due to the absence of egg production. In the un-irradiated pairs, field mosquitoes were 1.7 times less likely to produce viable offspring compared to laboratory mosquitoes with a greater probability of failing at both developing and laying eggs. Overall, according to Abbott’s formula [32], the percentage of radiation-induced sterility was about 80%.

## Discussion

### Insemination rate

The insemination rate was reduced by both radiation and rearing. The dose of radiation used in this study has been determined to be acceptable for a good sterility-competitiveness trade-off in *An. arabiensis* [22, 33]. The insemination rates were similar to those obtained by Helinski et al. [27] in large cages with a lower irradiation dose (70 Gy). Our irradiated field mosquitoes had a higher insemination rate than did their laboratory counterparts, suggesting that laboratory mass rearing might be an important factor affecting the way mosquitoes cope with irradiation effects when it comes to mating [34]. As for *An. albimanus*, irradiated pairs had the lowest insemination rate compared to other mating pairs [35] suggesting that both sexes are affected by irradiation and that both sexes are active in the mating process. Mating behavior, which leads to female insemination, occurs in swarms that consist mainly of males. Virgin females are known to join these swarms to form mating copulae in mid-flight [36]. Swarming behavior is very challenging for both males and females as the competition for a mate is strong. Males are numerous and they wait for females while swarming and sometimes chase each other inside the swarm. Once a female enters into the swarm, recognition between partners and probably the courtship behavior are based on the alteration of male and female wing beat frequencies until they converge into the same harmonic frequency [37]. To perform competitively, both males and females need high energy reserves as well as good flight and hearing abilities. Mosquitoes reared in the laboratory are not under high selective pressures compared to wild mosquitoes. Consequently, some of the laboratory mosquitoes, those that would have never survived or mated in the wild, may not possess enough reserves to compete for females in this very demanding behavior [38, 39]. Moreover, flight ability is also controlled by the thoracic muscles which are formed during metamorphosis. Pupae irradiation, as well as laboratory rearing, have been shown to alter flight muscles in numerous insects ([8] and references therein). In irradiated mosquitoes, the radiation may have altered their flight muscles so that they are less able to reach the wing beat that coincides with the same harmonic frequency as their partner and, as such, may be ignored during courtship thus preventing insemination. In addition, laboratory rearing does not select for flight capacity and this behavior may not be an advantage in small cages. Pheromone production (if any in *Anopheles* species [40]), which may be responsible for attractiveness and acceptance between the two sexes, might also be counter-selected or altered in both quality and quantity [8]. Similarly, hearing capabilities can be altered by irradiation. The Johnston’s organ located at the base of the antennae, which amplifies the air-born vibrations produced by wing beats, may be affected by irradiation. No study has yet demonstrated this but some studies have shown that irradiation and mass rearing alter the quality of vision in fruit flies [16] and in New World screwworms [17]. Consequently, it can be expected that other finely tuned senses may also be affected. Sperm production is also negatively affected by irradiation and may be a reason for a lower insemination rate [23, 24]. Altogether, the alteration of energy reserves, flight muscles, hearing capability, and both pheromone and sperm production, may be responsible for the drop in mating efficiency and for lower insemination rates.

### Egg prevalence and egg load

Fecundity was affected in several ways by both irradiation and laboratory rearing. First, irradiated females never produced eggs which is a common observation in irradiated female arthropods and was already observed in *An. arabiensis* [41]. Females are generally more radiosensitive than are males because of the later development of oocytes. Consequently, during irradiation, oocytes that have not yet produced eggs develop lethal mutations which inhibit egg development [42]. An exposition of 20-h-old pupae to a dose of 95Gy resulted in complete female sterilization. Second, field mosquitoes were slightly less likely to develop eggs. Here, differences between optimal rearing conditions and field larval site conditions may be responsible for this difference in female fecundity. Indeed, it has been shown that larval stresses have carry-over effects on adult fertility [43–45]. For example, stresses that affect food intake can impact the teneral reserves acquired during larval development. Teneral reserves are important for the production of the first egg batch in adults. Females that were well fed as larvae will need only one blood meal to produce their first egg batch while those with low teneral reserves will need two to three blood meals [46] which impacts fecundity during the first gonotrophic cycle. Finally, male irradiation also had an indirect negative effect on female fecundity. Indeed, irradiated males were less likely to inseminate females and non-inseminated females are known to be less fecund than inseminated ones ([47–49]; present study). This higher fecundity in inseminated females is triggered by the transfer of sex peptides and hormones from male accessory glands to the female atrium [47]. Nevertheless, as there was no difference in egg load and egg prevalence between un-irradiated inseminated females mated with irradiated or un-irradiated males, we can speculate that the production and transfer of these sex compounds are not directly affected by irradiation.

### Oviposition

Insemination by irradiated males did not affect oviposition. However, the mosquito strain had an impact on their decision to lay eggs. Indeed, while most of our laboratory females tended to lay their eggs all at once, field mosquitoes retained their eggs more frequently or did not lay at all. For organisms without parental care and with limited dispersal ability, oviposition site selection is a critical choice for reproductive success [50]. For mosquitoes, factors including larval competition, food quantity, predation or desiccation risk may be responsible for delayed oviposition or egg retention until more suitable conditions can be found [51–53]. Consequently, wild females are selected to be choosy thereby providing the best growing place for their progeny while laboratory females have been selected to oviposit on artificial substrates or in small containers. Thus, in our laboratory conditions, field females may be reluctant to oviposit in tap water, which is poor in nutrients, and retained their eggs more frequently than their laboratory counterparts.

### Hatching and larva viability

Only about 50% of the control females were fully sterilized when mated with irradiated males (no hatching). Nevertheless, within fertile female batches, only 15% of the eggs hatched. The radiation-induced sterility was about 80% which is very similar to results found in a previous study using the same radiation dose [22]. In addition, among females that produced fertile egg batches, only 50% of the batches produced at least one viable first-instar larva. This shows that induced mutations may be lethal at any developmental stages and, thus, that such studies should consider the entire larval development cycle until adult emergence to provide a more realistic picture of induced sterility [42]. To date, for mosquitoes, most interpretations are based on egg hatchability and thus probably underestimate the real efficiency of partially sterilizing radiation doses. A follow-up of larval mortality may help to optimize the competitiveness-sterility trade-off in irradiated males. Finally, no interaction between mosquito origin and radiation treatment was found for hatching rate or for larvae viability which means that neither of the two mosquito origins was better at coping with radiation than the other.

### Longevity

The effect of some stresses can be amplified when associated with a diet-based stress but even in our stressful design in which glucose was available only every other day, irradiation did not affect male longevity [3, 43]. However, field males survived longer than laboratory males. In the wild, the longer a male survives, the more it can mate. Longer longevity is therefore selected for. Conversely, laboratory-reared mosquitoes experienced different selective pressures which may keep weak males in the population. Therefore our results may be a consequence of laboratory rearing in which laboratory population longevity is impeded by the artificial selection of some weak males.

Our study includes two limitations. First, our laboratory mosquitoes were recently colonized (five generations) and we can speculate that larger differences may be expected with older colonies. Secondly, our experiments were conducted in small cages (insemination) and small vials (artificial substrate for oviposition) which are more favorable to laboratory-reared mosquitoes compared to field mosquitoes and make it difficult to extrapolate as to how laboratory mosquitoes will respond once released into the wild.

## Conclusions

Overall, our study reveals the different impacts of mosquito strain and irradiation on reproductive processes. The different steps in progeny production, from insemination to larval survivorship, were not affected to the same magnitude or in the same way for laboratory and wild mosquitoes. Despite these differences, the final potential reproductive success of both irradiated, laboratory-reared and irradiated, field mosquitoes were similar (10.2 *vs* 8.33%, respectively; Fig. 8). This highlights the need to consider as many fitness traits as possible when evaluating the efficiency of the sterile insect technique. As parental effects may also play a role in the reproductive success of the progeny, such studies should be extended to examine progeny fecundity and fertility. Finally, irradiated females never produced eggs. If irradiated females are released they will not contribute to maintaining population density. However, they may still be potential vectors of malaria. Because current methods of sexing are not fully efficient [54], further studies are needed to determine if irradiated females are competent vectors.

## Additional files

Additional file 1: **Figure S1.** Effect of insemination on egg number in un-irradiated females. The number of eggs is the sum of eggs laid or retained in the ovaries per female. The numbers in the bars indicate sample size. *P < 0.05. **Figure S2.** Effect of insemination on oviposition rate in un-irradiated females (i.e. females that laid at least one egg). The numbers in the bars indicate sample size. ***P < 0.001. **Table S1.** Mating combinations. **Table S2.** Statistical models used in data analyses. (DOCX 54 kb)

## Abbreviations

L:D: Light:dark
PCR: Polymerase chain reaction
RH: Relative humidity
SIT: Sterile insect technique
