# Supplementary material for: Impact of irradiation on the reproductive traits of field and laboratory An. arabiensis mosquitoes
Source: Parasit Vectors. 2018 Dec 17;11:641. doi: 10.1186/s13071-018-3228-3 (PMC6296153; doi:10.1186/s13071-018-3228-3)
Supplement: Supplementary file 1 — Figure S1. Effect of insemination on egg number in un-irradiated females. The number of eggs is the sum of eggs laid or retained in the ovaries per female. The numbers in the bars indicate sample size. *P < 0.05. Figure S2. Effect of insemination on oviposition rate in un-irradiated females (i.e. females that laid at least one egg). The numbers in the bars indicate sample size. ***P < 0.001. Table S1. Mating combinations. Table S2. Statistical models used in data analyses. (DOCX 54 kb) [file 13071_2018_3228_MOESM1_ESM.docx]

**Impact of irradiation on the reproductive traits of field and laboratory *An. arabiensis* mosquitoes**

Serge B. Poda^1,2^, Edwige Guissou^1,2^, Hamidou Maïga^1^, Sévérin Bimbile-Somda^1,3^, Jérémie Gilles^3^, Jean-Baptiste Rayaisse^4^, Thierry Lefèvre^1,2^, Olivier Roux^1,2†*^ and Roch K. Dabiré^1†^

^1^Institut de Recherche en Sciences de la Santé (IRSS), 01 BP 545 Bobo-Dioulasso 01, Burkina Faso.

^2^MIVEGEC, IRD, CNRS, University of Montpellier, Montpellier, France.

^3^Insect Pest Control Laboratory, Joint FAO/IAEA Division of Nuclear Techniques in Food and Agriculture, Vienna, Austria.

^4^Centre International de Recherche-Développement sur l’Elevage en zone Subhumide (CIRDES), 01 BP 454 Bobo-Dioulasso 01, Burkina Faso.

*Correspondence: olivier.roux@ird.fr

†Olivier Roux and Roch K. Dabiré contributed equally to this work.

**Additional file 1: Figure S1. Effect of insemination on egg number in un-irradiated females.** The number of eggs is the sum of eggs laid or retained in the ovaries per female. The numbers in the bars indicate sample size. * P<0.05.

**Additional file 1: Figure S2. Effect of insemination on oviposition rate in un-irradiated females.** (*i.e.* females that laid at least one egg). The numbers in the bars indicate sample size. *** P<0.001

**Additional file 1: Table S1.** Mating combinations.

| **Couple designation** | **Mating combinations** | | |
| --- | --- | --- | --- |
|  | **Males** |  | **Females** |
| Un-irradiated pairs | Un-irradiated | x | Un-irradiated |
| Irradiated pairs | Irradiated | x | Irradiated |
| Irradiated male pairs | Irradiated | x | Un-irradiated |
| Irradiated female pairs | Un-irradiated | x | Irradiated |

**Additional file 1: Table S2**: **Statistical models used in data analyses.**

| **Mosquito traits** | **Response variable** | **Maximal model** | **R subroutine [error structure]** | **N** |
| --- | --- | --- | --- | --- |
| **Emergence** |  |  |  |  |
|  | **Emergence rate** | strain * treatment + (cup) | glmer[b] | 5420 |
| **Insemination** |  |  |  |  |
|  | **Insemination rate** | strain * treatment + (cage) | glmer[b] | 561 |
| **Fecundity** |  |  |  |  |
|  | **Egg prevalence** | strain * treatment * insemination + (cage) | glmer[b] | 296 ^1^ |
|  | **Egg load** | strain * treatment * insemination + (cage) | glmmadmb[nb] | 235 ^2^ |
|  | **Oviposition rate** | strain * treatment + (cage) | glmer[b] | 208 ^3^ |
|  | **laid egg rate** | strain * treatment + (cage) | glmer[b] | 181 ^3^ |
|  | **Hatching rate** | strain * treatment + (cage) | glmer[b] | 137 ^4^ |
|  | **Hatching proportion** | strain * treatment + (cage) | glmer[b] | 109 ^5^ |
| **Fertility** |  |  |  |  |
|  | **Larval prevalence** | strain * treatment + (cage) | glmer[b] | 97 ^5^ |
| **Longevity** |  |  |  |  |
|  | **Day of death** | strain * treatment + (cage) | coxme | 1472 |

"**Maximal model**" gives the complete set of explanatory variables and their interactions included in the model.

^1^ Includes only un-irradiated females (irradiated females did not produce eggs).

^2^ Includes only gravid un-irradiated females. Treatment was: irradiated and un-irradiated males, here and in the following models.

^3^ Includes only gravid and inseminated females (non-inseminated females did not lay eggs).

^4^ Includes all batches of laid eggs.

^5^ Includes only egg batches in which there was hatching (at least one egg hatched).

* included main effects and one way interactions only.

() variable fitted as random effect.

[ ] error structure used (b: binomial errors, nb: negative binomial errors).

N=number of pupae, mosquitoes or egg batches included in the analysis.
